# Effective osimertinib treatment in a patient with discordant T790 M mutation detection between liquid biopsy and tissue biopsy

**DOI:** 10.1186/s12885-018-4222-z

**Published:** 2018-03-21

**Authors:** Isa Mambetsariev, Lalit Vora, Kim Wai Yu, Ravi Salgia

**Affiliations:** 1 Department of Medical Oncology and Therapeutics Research, City of Hope Comprehensive Cancer Center and Beckman Research Institute, 1500 E Duarte Rd, Duarte, CA 91010-3000 USA; 2 Department of Diagnostic Radiology, City of Hope Comprehensive Cancer Center and Beckman Research Institute, Duarte, CA 91010 USA; 3 Department of Pharmacy Services, City of Hope Comprehensive Cancer Center and Beckman Research Institute, Duarte, CA 91010 USA

**Keywords:** EGFR T790 M-positive NSCLC, Osimertinib, Progression, Liquid biopsy, Dose, Case report

## Abstract

**Background:**

We report the successful treatment of the patient with osimertinib 80 mg/day following disease progression and a discordance in the detection of a mechanism of resistance epithelial growth factor receptor (EGFR) T790 M between liquid biopsy and tissue biopsy methods.

**Case presentation:**

A 57-year-old Hispanic male patient initially diagnosed with an EGFR 19 deletion positive lung adenocarcinoma and clinically responded to initial erlotinib treatment. The patient subsequently progressed on erlotinib 150 mg/day and repeat biopsies both tissue and liquid were sent for next-generation sequencing (NGS). A T790 M EGFR mutation was detected in the blood sample using a liquid biopsy technique, but the tissue biopsy failed to show a T790 M mutation in a newly biopsied tissue sample. He was then successfully treated with osimertinib 80 mg/day, has clinically and radiologically responded, and remains on osimertinib treatment after 10 months.

**Conclusions:**

Second-line osimertinib treatment, when administered at 80 mg/day, is both well tolerated and efficacious in a patient with previously erlotinib treated lung adenocarcinoma and a T790 M mutation detected by liquid biopsy.

## Background

Non-Small Cell Lung Cancer (NSCLC) is a devastating disease and is the leading cause of cancer-related death worldwide. However, the treatment options for NSCLC have evolved dramatically in the last decade and NSCLC has become instrumental in advancing the new age of personalized medicine where standard platinum doublet chemotherapy is substituted by tyrosine kinase inhibitors (TKIs) when patients are tested and diagnosed with actionable mutations. The specificity of the disease and the histological differences in individual cancer types, such as lung adenocarcinoma, no longer dictate the clinical outcome and potential treatment options, but it is really the omic-architecture of individual patients that becomes the driver of treatment for targeted therapy as well as immunotherapy. The genomic layout of adenocarcinomas is continuously being redefined with a myriad of genetic alterations, such as mutations in EGFR, MET, and BRAF, translocations in ALK and ROS1, detected and present in the TCGA dataset [1]. For example, approximately 10% of patients with NSCLC in the US have tumor involving the epidermal growth factor receptor (EGFR) somatic activating mutations [2]. Exon 19 deletion mutations and single-point substitution mutation L858R in exon 21 are considered “classic” mutations that are sensitive to EGFR TKIs and account for 90% of all EGFR mutations in NSCLC [3, 4]. The presence of these mutations in select NSCLC patients showed dramatic TKI response rates (RRs) of 68% with a mean progression-free survival (PFS) and time to progression of 12 months [5–7].

Based on this evidence, EGFR testing and EGFR TKIs have become a staple of lung cancer clinical treatment and since then the College of American Pathologists, International Association for the Study of Lung Cancer, and the Association for Molecular Pathology have standardized the testing guidelines for selection of lung cancer patients for EGFR inhibitors as well as the methods of testing, such as real-time polymerase chain reaction (PCR) and next-generation sequencing (NGS) [8]. Nevertheless, almost all patients who initially respond to EGFR inhibitors in NSCLC eventually develop acquired resistance (AR). So far, it is known that a secondary T790 M mutation in exon 20 of the EGFR gene accounts for approximately 50% of cases of AR, alongside other less understood mechanisms of resistance [9]. The FDA has recently approved Osimertinib for the treatment of patients with metastatic T790 M mutation-positive non-small cell lung cancer (NSCLC) based on the evidence of the AURA3 trial where progression-free survival benefit was observed in both progressive NSCLC following first-line EGFR TKI therapy and EGFR T790 M mutation-positive NSCLC identified by the cobas EGFR mutation test [10, 11]. A recent study on the detection of T790 M by Tumor Biopsy versus Noninvasive Blood-based methods showed that the overall T790 M mutation positive rate was approximately 50% consistent with previous biopsy series [12]. In this case report, we present a male patient diagnosed with EGFR positive lung adenocarcinoma that partially responded to erlotinib, but eventually progressed. Upon progression the patient’s blood and tissue samples were tested using NGS. The tissue biopsy test failed to detect a T790 M mutation, while the liquid biopsy test successfully showed a T790 M resistance mutation present in the patient’s blood. The patient was treated with osimertinib with notable clinical and radiological response. The patient continues to be treated successfully with osimertinib and will remain on osimertinib treatment after 6 months.

## Case presentation

A 57 year old Hispanic male who smoked for one year initially presented with lower back pain that eventually progressed to increasing upper back pain. An orthopedic surgeon performed an MRI scan which revealed multiple metastases to the thoracic spine. An initial CT scan revealed a right lung mass and a subsequent bronchoscopy revealed a non-small cell lung cancer consistent with poorly differentiated adenocarcinoma. For first line therapy, he received one cycle of carboplatin and docetaxel which was tolerated well with only mild nausea. He completed four cycles of chemotherapy and a pathology report identified an EGFR exon 19 deletion. The patient started on erlotinib, an EGFR inhibitor, at 150 mg capsule per day taken by mouth without food or on an empty stomach 1 h before or 2 h after food. Patient was also advised to avoid sunlight while on therapy due to skin toxicity. He tolerated it well and had clinically responsive disease as well as a decrease in primary mass from 5 cm to 2.8 cm on CT scan for over a year until he had started developing worsening back pain and complications caused by papulopustular lesions as well as paronychia and xerosis. He also developed a Klebsiella folliculitis which necessitated discontinuation of the trimethoprim-sulfamethoxazole that he had been taking for the acneiform lesions and a brief course of ciprofloxacin that had led to the resolution of the papulopustular eruption.

He also started to have persistent elevation of carcinoembryonic antigen (CEA) level from 5.4 ng/ml rising steadily to 13.4 ng/ml and eventually rising exponentially to 24.3 ng/ml but continued on erlotinib, dose reduced to 100 mg one capsule per day, due to stable MRI scans. Patient was evaluated for a possible surgery on the spine but was unfortunately not a surgical candidate. A CT of the chest with and without contrast reported significant primary site progression of bilateral varying size small lung nodular lesions, consistent with metastatic. The right infrahilar nodular mass also increased from 2.1 × 1.5 cm to 2.8 × 2.4 cm. Clinically he was progressing and he underwent a fine needle biopsy on the right lung mass and pathology reported moderately differentiated metastatic adenocarcinoma. Tissue was sent for molecular testing via next generation sequencing with CLIA-certified Foundation Medicine multi-gene assay and a blood draw was performed for liquid biopsy utilizing the Guardant 360 platform, which are both comparable to the cobas EGFR tissue test and plasma ctDNA test in the AURA3 trial. While awaiting the results, erlotinib was stopped and he was given one cycle of carboplatin AUC 5 and pemetrexed 500 mg/m2. Soon afterward liquid biopsy report returned and was shown to be positive for a T790 M mutation that is a mechanism of acquired resistance to EGFR tyrosine kinase inhibitor (TKI) therapy. Liquid biopsy utilizing the Guardant 360 platform also detected ten other mutations; EGFR exon 19 deletion, TP53 R196Q mutation, FGFR3 L406R mutation, VHL S65A mutation, RHOA E47K mutation, APC D1512N mutation, NTRK1 T360 T mutation, PDGFRA I497I mutation, FGFR2 E767K mutation, BRCA1 E962K mutation.

**Fig. 1:**
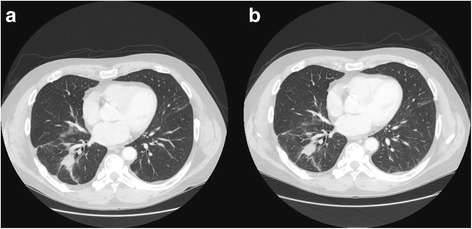
Chest CT Scans of Patient on Osimertinib Treatment. **a** Chest CT Scan Prior to Osimertinib Treatment. **b** CT Chest w/Abdomen wwo/Pelvis w Contrast Scan Post-Osimertinib Treatment

Based on the T790 M mutation he was started on osimertinib, at 80 mg tablet per day taken by mouth, and tolerated it well. A few weeks into the osimertinib treatment the tissue biopsy test reported five mutations, CDK4 amplification, MDM2 amplification, FRS2 amplification, GLI1 amplification as well as the EGFR exon 19 deletion and nine variants of unknown significance; EP300 M2372 V, GNAS P345R and P349_I357del, IRF4 A341V, MED12 Q2120_Q2121 > HQQQQQ, MET D1373H, MLL A53V, SMARCA4 A1186_Q1187del, SOX9 A481T, SPTA1 R1077C. However, tissue biopsy test did not detect a T790 M mutation showing discordance between the tissue biopsy and the liquid biopsy. He continued on osimertinib treatment and a Chest CT (Fig. 1) scan ten months into the treatment showed a decrease in the right lower lobe mass and numerous bilateral pulmonary nodules had either significant decrease or resolved. There were also stable widespread osseous metastases. This was noted to be a partial response and it was decided that the patient will continue on osimertinib. He has tolerated osimertinib well with only symptoms of a mild rash and low platelets which quickly stabilized without dose adjustment.

## Discussion and conclusions

**Fig. 2:**
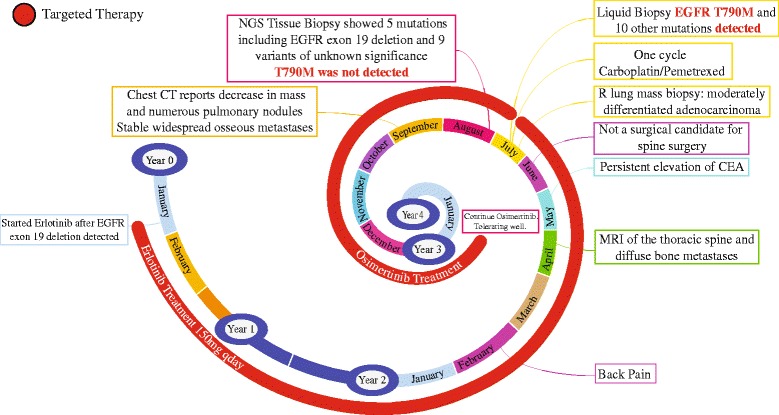
Timeline of Patient’s Oncologic History

In this study, the treatment plan for the patient could have been potentially undermined by the NGS technology and lack of standardization of detection methods for EGFR mechanisms of resistance. This shows that tumor heterogeneity plays an important role not only therapeutically but is reliant on clinical efficacy to do what is beneficial for the patient based on the evidence of a blood-based T790 M detection. Our case report reflects not only a molecular switch within the tumor, but also highlights the discordance in one sample and not the other. As can be appreciated, the patient initially had L858R EGFR abnormality and responded to erlotinib. However, upon clinical and radiological progression, the tumor biopsy of the lung lesion still showed the original L858R EGFR abnormality, but did not detect the classic T790 M mutation of acquired resistance. Interestingly, the liquid biopsy revealed the classic T790 M EGFR mutation along with the original EGFR mutation. The absence of a T790 M mutation in tumor tissue may be explained by the limited tissue available from the lung as well as inter- and intra-tumor heterogeneity where the tissue biopsy does not give a full viewpoint of the entire tumor or the genetic heterogeneity of metastatic disease. Meanwhile the detection of a T790 M mutation in the liquid biopsy may be due to the nature of metastatic disease and the diverse circulating tumor DNA population that represents the overall mixed heterogeneity of the disease rather than limited to a small tissue sample from a single site. Based on this, we were able to make intelligent clinical decision to start osimertinib and the patient is responding (thus far, up to 10 months and continuing). Figure 2 summarizes the patient’s oncologic history as a timeline.

In the past, we had poorly utilized biomarkers such as CEA and CA125 to determine the response or progression to therapy [13]. More recently, we have begun to determine the role of tumor tissue genomic biomarkers and circulating biomarkers. The molecular diagnostic field has come far over the past few years and the advent of liquid biopsies offers an opportunity to circumvent invasive tissue biopsies at the time of each disease progression. Liquid biopsies offer flexibility and repeatability for the patient that tissue biopsies do not due to high costs, high morbidity, or lack of available tissue. Though the list of currently available approved targeted therapies remains limited, only recently the FDA granted accelerated approval to the EGFR TKI osimertinib for patients with advances T790 M mutation-positive NSCLC based on the evidence of two-single arm studies with RRs between 57–61% [14, 15]. Consequently, in a recent study, we showed that NSCLC patient assessment of targeted therapies using commercially available ctDNA assays had a high concordance of 80% between paired tissue and blood for truncal oncogenic drivers and patients with biomarkers identified in plasma had expected progression free-survival (PFS) [16]. Though there may be no difference in progression-free survival between the liquid-positive and tissue-positive groups, there is still a necessity to consider this as a novel molecular diagnostic tool that requires less intervention and can possibly in the future be widely used to treat tumor heterogeneity over time in advanced stage NSCLC [17]. Therefore, we would recommend that analysis of biomarkers be routinely considered in mechanisms of resistance as well in the initial molecular diagnosis of advanced stage adenocarcinoma of the lung [17]. This case reflects the discordance between tissue and liquid biopsy. As we go forward in decision making, we might have to utilize both diagnostics to arrive at a meaningful clinical decision.

## Abbreviations

AR: Acquired resistance
CEA: Carcinoembryonic antigen
CT: Computed tomography
ctDNA: Circulating tumor DNA
EGFR: Epithelial growth factor receptor
MRI: Magnetic resonance imaging
NGS: Next-generation sequencing
NSCLC: Non-small cell lung cancer
PCR: Polymerase chain reaction
PFS: Progression free survival
RR: Response rate
TKI: Tyrosine kinase inhibitor
